# Extreme heat exposure of host plants indirectly reduces solitary bee fecundity and survival

**DOI:** 10.1098/rspb.2024.0714

**Published:** 2024-06-19

**Authors:** Jenna Walters, McKenna Barlass, Robin Fisher, Rufus Isaacs

**Affiliations:** ^1^ Department of Entomology, Michigan State University, East Lansing, MI 48824, USA; ^2^ Program in Ecology, Evolution, and Behavior, Michigan State University, East Lansing, MI 48824, USA

**Keywords:** climate change, extreme heat, native bees, crop pollination, pollinators, solitary bees

## Abstract

Extreme heat poses a major threat to plants and pollinators, yet the indirect consequences of heat stress are not well understood, particularly for native solitary bees. To determine how brief exposure of extreme heat to flowering plants affects bee behaviour, fecundity, development and survival we conducted a no-choice field cage experiment in which *Osmia lignaria* were provided blueberry (*Vaccinium corymbosum*), phacelia (*Phacelia tanacetifolia*) and white clover (*Trifolium repens*) that had been previously exposed to either extreme heat (37.5°C) or normal temperatures (25°C) for 4 h during early bloom. Despite a similar number of open flowers and floral visitation frequency between the two treatments, female bees provided with heat-stressed plants laid approximately 70% fewer eggs than females provided with non-stressed plants. Their progeny received similar quantities of pollen provisions between the two treatments, yet larvae consuming pollen from heat-stressed plants had significantly lower survival as larvae and adults. We also observed trends for delayed emergence and reduced adult longevity when larvae consumed heat-stressed pollen. This study is the first to document how short, field-realistic bursts of extreme heat exposure to flowering host plants can indirectly affect bee pollinators and their offspring, with important implications for crop pollination and native bee populations.

## 1. Introduction

The warming climate is a key driver of insect population declines [1–4], yet the various ways in which these changes affect insects are still being elucidated. Acute bouts of extreme heat are becoming more frequent and intense [5], negatively affecting plants and the insects that they depend on, including bees and their pollination services [6–10]. Studies investigating the direct effects of extreme heat on bees or plants offer important insights into the consequences of heat on their physiology [11–15], capacity for acclimation [16–23] and reproductive potential [12,24–27]. However, when extreme heat events occur in the environment, bees and their host plants both endure heat stress, potentially resulting in compounding, interactive ramifications for these organisms. Despite this, investigations intersecting the effects of extreme heat on bee–plant interactions are limited [28–30], fragmenting our understanding of heat stress repercussions on pollination systems. In plant–pollinator networks, synchrony is critical for their success and survival, and extreme heat can disrupt the timing of these interactions [31–34]. While phenological synchrony is important to understand in the context of climate change, few studies have explored whether heat stress also affects the synergy between bee pollinators and their host plants. For example, there is little information on whether heat-stressed plants adequately support the dietary needs of their bee pollinators. As bouts of extreme heat continue to intensify [5], it is critical to broaden our understanding of how indirect heat stress affects bee–plant interactions.

Plant reproductive development, particularly male gametophyte (i.e. pollen) development and performance are the most sensitive development stages to heat stress [14,20,24,35–37]. When extreme heat occurs during floral development, maturation and/or dehiscence, it can have compounding adverse consequences, including degradation of the tapetum, failure to release microspores, altered metabolism and transport of nutrients in pollen, reduced pollen viability, poor anther dehiscence and failure to release pollen [24,36,38–40]. When heat inhibits nutrient sequestration in developing pollen, concentrations of carbohydrates, proteins, lipids and amino acids can be reduced or altered [24,38,39,41,42]. Many of these nutrients drive reproductive processes including pollen germination and tube growth, which are necessary for fertilization, so this depletion of nutrients can reduce pollen quality, performance and subsequent reproduction [14,24,37,40,42]. Some researchers have recently hypothesized that these heat-induced pollen nutrient reductions could also negatively affect bees, as these insects rely on the nutrients present in pollen for their diets [10,29,41,42]. However, no studies have confirmed the connection between heat-stressed pollen and bee nutrition, requiring further research. Extreme heat may also reduce the production and release of pollen [24,26,37,43], limiting bee access to floral rewards. These changes could lead to nutritional stress with important consequences for bee fecundity, behaviour and development [44–47]. Subsequent offspring can also be affected as reduced quantity and quality of pollen provisions may lead to altered developmental timing, shifts in sex ratios, reduced body size and higher rates of mortality [48–51]. These adverse effects on bees could limit pollination services, affecting the reproduction of wild plants [34] and crops [10,33]. Despite the potential ramifications, there is limited cross-disciplinary understanding of the effects of extreme heat on bees, plants and their interactions.

While most research exploring the impacts of extreme heat on bee pollinators has focused on social bees, studies suggest greater sensitivity to extreme heat in solitary bees [15], such as *Osmia* bees. *Osmia lignaria* Say (Hymenoptera: Megachilidae) is a solitary, polylectic, stem-nesting mason bee native to North America. Wild and managed *O. lignaria* are important pollinators of spring-blooming wildflowers and crops [52–55]. However, recent studies suggest *O. lignaria* populations are in decline in the US, driven by increased competition and disease prevalence [56–58] as well as pesticide exposure [59–61]. Direct nutritional and heat stress can also negatively affect these bees [31,47,50,62–66], but there is limited understanding of the indirect effects of extreme heat on *Osmia* bees and their offspring. Given the evidence for declining populations and the importance of their pollination services, it is imperative to identify and mitigate stressors affecting wild bees.

Northern highbush blueberry is a spring-blooming perennial fruit crop native to North America that is highly dependent on wild pollinators and is visited by *O. lignaria* [67,68]. In temperate regions where this crop grows, spring temperatures are typically moderate, but extreme heat events have become more common [5,69]. In 2018, Michigan blueberry growing regions endured temperatures exceeding 35°C for several hours during bloom, associated with 30–50% yield reductions [70]. Blueberry pollen exposed to extreme heat, even for 4 h, can drastically and irreversibly inhibit performance, potentially limiting fertilization and yields [70]. Lacy phacelia is a herbaceous flowering plant native to North America, visited by *O. lignaria* in the wild and semi-field experiments [51,71,72]. While no studies to our knowledge have assessed the effects of heat stress on blooming phacelia, optimum temperatures are reported to be between 23 and 30°C [22,73] with high seedling mortality following acute heat stress [74,75]. White clover is a herbaceous flowering plant native to Europe and Central Asia, introduced and widely distributed across North America. It is commonly found blooming near blueberry fields and frequently visited by *O. lignaria* [67,76]. Brief heat exposure can cause abiotic stress in white clover [77], resulting in fewer inflorescences [78] or vegetative tissue loss and mortality [79]. While crop plants have received greater attention, many plants enduring high temperatures (>35°C) during floral development, even for a few hours, experience adverse repercussions on pollen quality, performance and subsequent plant functioning [20,24,35–37].

As extreme heat events become more common and intense, and bee declines continue to escalate, further research is required to provide a broader understanding of how native solitary bees are affected by extreme heat. We investigated the indirect effects of extreme heat on *O. lignaria* and their offspring by releasing females in field cages to forage on blueberry, phacelia and white clover exposed to extreme heat (37.5°C for 4 h) or control conditions (25°C for 4 h) during bloom. Bees were observed during foraging and egg laying, and their offspring were monitored *in vitro* from eggs to adults. The study was designed to determine whether: (i) heat-stressed host plants affect *O. lignaria* maternal foraging and fecundity; (ii) the development and survival of larvae are affected by consuming pollen from heat-stressed plants; and (iii) the emergence, survival, body size and sex ratio of adult progeny are affected by heat-stressed larval diets. We expected female *O. lignaria* bees provided with heat-stressed plants to have similar foraging rates but reduced fecundity. We also predicted altered larval development and reduced survival for offspring-fed pollen from heat-treated plants. For adult offspring who consumed heat-stressed diets as larvae, we hypothesized reduced emergence, survival and body size altered timing of emergence and longevity and male-dominated sex ratios.

## 2. Material and methods

### (a) Biological material

This study used three different host plants: blueberry, lacy phacelia and white clover. Dormant 2-year-old ‘Bluecrop’ blueberry bushes were purchased in winter (Hartmann Nursery, Grand Junction, MI and DeGrandchamp Farms, South Haven, MI) and immediately placed in dark cold storage (2°C) until 1200 chilling hours had accumulated. When needed, bushes were moved to a greenhouse at 22 ± 5°C and 16 : 8 light : dark (L : D) cycle. Plants were watered regularly, and soil pH was monitored every 3–4 weeks to ensure it was < 6.0 [80]. When necessary, bushes were treated with an acidifier (Jobe’s Organics, Easy Gardener Products, Waco, TX) and a fertilizer (Osmocote, The Scotts Company, Marysville, OH) following the manufacturer label. Phacelia and white clover seeds were purchased from L.A. Hearn Company (King City, CA), and sown in 1 litre plastic pots with a mixture of potting soil (Michigan Grower Products, Inc., Galesburg, MI) and field soil at a 50 : 50 ratio, ensuring optimal soil moisture for growth [81–83] and kept in a greenhouse (22 ± 5°C, 16 : 8 L : D). Approximately 20 pots of phacelia and clover were sown each week (January–April 2022) to ensure sufficient blooming plants for experiments.

### (b) Exposing host plants

At approximately 25% bloom, plants were randomly assigned to control temperature (CT: 25°C for 4 h) or high temperature (HT: 37.5°C for 4 h) conditions. Plants were exposed at 25% bloom because this was the approximate blueberry development stage in 2018 during the extreme heat event described above, it allowed for a wider breadth of floral development stages exposed to heat (from developing buds to open blooms) and this maximized the duration of bloom (and thus floral resources) available to bees. Phacelia and white clover were also exposed at 25% bloom to ensure consistency of heat stress exposure. The temperature for the HT regime was selected to mimic recently experienced acute extreme heat events where daily maximum temperatures were > 20°C hotter than historical daily maximums in 2018, exceeding 35°C for 4 h during blueberry bloom (Global Historical Climatology Network). In general, heat stress occurs when temperatures are 10–15°C above ambient [84]. Previous studies have shown that short exposure (<5 h) to >35°C can negatively affect pollen performance in blueberry [70] and other plant species [14,20,36]. While several studies discussed above have found a relationship between altered pollen nutrition and poor pollen viability following heat stress, we re-emphasize that no studies have reported a connection to nutrition availability for bees. However, we and others hypothesize that such reductions in pollen viability driven by nutritional deficits may also have negative consequences for bee nutrition [10,29,41].

Environmental growth chambers were set to CT and HT conditions and temperatures were monitored every 30 min using HOBO temperature loggers (Onset Computer Corporation, Bourne, MA). Plants were assigned in pairs such that multiple plants received the HT and CT treatment conditions at the same time in each respective chamber. After exposure to the appropriate temperature regime, plants were immediately moved to the field cages as described below.

### (c) *Osmia lignaria* bees for experiments

Male and female *Osmia lignaria* cocoons were purchased from Meyer Bees (Meyer Bees, Minooka, IL) in March 2022. Cocoons were kept dormant (no light, 4°C, 60% RH) until needed for experiments [59,85]. In June 2022, we began moving cocoons to emergence conditions. We placed 20 male and 10 female cocoons in 0.3 × 0.3 m plastic mesh cages (BioQuip, Rancho Dominguez, CA) next to a window for natural light, maintained at 20–22°C in three cohorts on 10, 15 and 20 June 2022. Bees took 3–5 days to emerge and were provided sugar water (50% sucrose) *ad libitum* via a dental wick in a glass flask which was replaced every week. Once emerged, observations were made daily to ensure successful mating. Bees typically mated 2–3 days following emergence. Mated females were marked on their thorax using a uniquely coloured non-toxic paint pen (Mitsubishi Pencil Co., Tokyo, Japan), then moved to a separate cage maintained under the same conditions until released in field cages. Bees were marked to allow for individual behaviour assessments in field cages. Once sufficient floral resources were available, three mated females were released in each cage. Each bee was similar in size and age (3–4 days old). After all cages received their initial three bees, some cages received replacement bees if one was found dead or missing within 1 day of its release or if bees died and failed to produce eggs. Replacement bees were released in two cages under these criteria to maintain activity and egg laying.

### (d) Cage experimental design

Using a no-choice design, we measured the foraging and egg laying of *O. lignaria* in field cages provided with HT or CT host plants. At the Entomology Research Farm (East Lansing, MI), eight 3.7 × 1.8 m mesh field cages (BioQuip, Rancho Dominguez, CA) were constructed, spaced 2 m apart. Field cages were randomly assigned to a temperature treatment, where four cages received HT-treated plants and four cages received CT-treated plants. Ambient air temperature averaged 25°C throughout the study at a nearby weather station (enviroweather.msu.edu), ranging from 18 to 31°C.

Within each field cage, a nest box was placed on the opposite end from the opening, facing east. Each nest box, adapted from [47], consisted of ten 170 × 180 mm pieces of wood stacked inside a plywood box with an open face and slanted roof, mounted on two metal poles, at 1.2 m height. In each nesting plank, 10 cavities were routered (Ryobi, Anderson, SC), each 10 mm wide and deep, 160 mm long and spaced 7 mm apart, providing 100 blind-ended cavities in each cage. Clear acetate sheets cut to the same dimensions were placed on top of each plank, allowing observation and extraction of eggs and provisions with minimal disturbance. In front of each nest box, we created a 0.3 × 0.6 m bare patch of moist soil to allow bees access to mud for their nest construction.

Cages were randomly paired in four replicate blocks, with one cage from both temperature groups in each block. Cages were paired to match timing of plant and bee placement while also accounting for the capacity of growth chambers to expose plants to treatments. This allowed for a consistent distribution of newly exposed blooming plants, ensuring sufficient resources for female bees and their offspring. Following heat exposure, plants were immediately moved to field cages (~5 km away). The exposure and initial placement of all host plants occurred on the same day within paired blocks, between 14 and 29 June 2022. Bees were released in cages within 2–3 days of initial blueberry plant placement, between 16 and 27 June 2022. Phacelia and clover plants were added 0–5 and 2–13 days, respectively, after bee releases and bees were able to forage on these plants the same day of plant exposure and placement. To maintain floral densities, subsequent additions of newly exposed plants (and removal of non-blooming plants) occurred from 22 June to 26 July 2022, with 5, 3 and 3 days between replacements for blueberry, phacelia and clover, respectively. All replacement plants were moved to field cages immediately after their temperature treatment. In each cage, 26–30 blueberry, 10–15 phacelia and 6–13 clover plants were provided throughout the experiment. We counted the number of open flowers on each plant, typically on the same day as behaviour assessments, or within 2–3 days, throughout the experiment. Plants were placed in the cages in rows, with blueberry placed on the outer rows (closest to the cage walls) and phacelia and clover placed on inner rows, between the blueberries. This ensured visibility of the nest box and floral visitation observations. Foraging observations and floral assessments occurred in 2022 from 17 June to 29 July in Blocks 1 and 2, from 23 June to 25 July in Block 3, and from 28 June to 25 July in Block 4.

### (e) Behaviour assessments

Bees were released the same day within blocked pairs, between 16 and 27 June 2022. To allow time to adjust to new conditions, foraging observations began the day following release. Bee foraging observations occurred from 17 June to 29 July 2022. Observations were 30 min per cage, conducted 3–5 days per week, typically between 9:00 and 15:00 when weather conditions were suitable. The order in which field cages were assessed was randomized daily. For each individual bee, we recorded the plant visited and the number of floral visits per plant. Floral visits were recorded when bees were actively collecting nectar or pollen. All bees (in each cage) were assessed by a single observer sitting inside the cage by the entrance, opposite the nest box. These observations also allowed the identification of nest cavities being provisioned by *Osmia* bees. Field cage observations concluded in early August once all bees died, and the flowers were depleted.

### (f) *Osmia* development assays

Eggs and pollen provisions were collected from nest boxes typically the same day the eggs were laid, or within two days. Egg collection occurred at night, minimizing disturbance and potential damage to mother bees and eggs. We used sterilized featherweight forceps (BioQuip, Rancho Dominguez, CA) to place each egg and associated pollen provision in a 1.5 ml centrifuge tube with a piece of sterilized tin foil placed inside, holding them upright. These were transferred to the lab and each pollen provision was weighed on a precision balance (Mettler Toledo, Columbus, OH), accurate to 1 mg. Pollen provisions and eggs were transferred to sterilized 48-well cell culture plates with 10 mm diameter cells, adapted from [59,86]. Each well plate received 3–7 eggs, with at least one egg from both temperature treatments. Plates were kept in a dark environmental growth chamber (22°C + 60% RH) and were removed for brief periods every 1–2 days to assess larval survival and growth stage using a dissection microscope. We recorded development stages as egg/1st instar, 2nd/3rd instar, 3rd/4th instar, 5th instar, cocoon spinning or fully spun cocoon [87]. Eggs were considered alive if they appeared undamaged (no holes, not deflated), and larvae were considered alive if they were moving or if spiracles on the side of the body were dilating, indicating breathing. Developing offspring were maintained at 22°C + 60% RH for 120 days, then moved to pre-wintering conditions (21 days: 14°C + 60% RH), wintering conditions (120 days: 4°C + 60% RH) and finally emergence conditions (22°C + 60% RH). The pre-winter weight of cocoons was recorded using a precision balance immediately before moving them to winter conditions.

### (g) Adult emergence and survival assessments

The day cocoons were moved to emergence conditions, the post-winter weight was recorded to determine the change in weight following overwintering. Daily checks were conducted to determine the timing of emergence and survival of adult bees. Emerged bees were kept in well plates without food provisions to determine their longevity. Upon the first day of adult emergence, bees were weighed and placed back in well plates. Once bees died, they were removed, sexed and the intertegular distance was recorded as a proxy of body size and expected flight capacity [88]. All emerged progeny in this study were identified as males.

### (h) Data analysis

All statistical analyses were conducted in R (R v. 4.2.3) [89]. We used generalized linear mixed-models (GLMM) (‘lme4’ package) [90] and mixed effects Cox models (‘coxme’ and ‘survival’ packages) [91,92]. Decisions on final models were based on the nature of the data taken, meeting the assumptions of the model distribution, assessing the lowest AIC model scores (‘bbmle’ and ‘stats’ packages) [89,93], the model deviance residuals (‘base’ package) [89] and other model performance metrics (‘performance’ package) [94]. GLMM model assumptions were checked by assessing scaled residuals using ‘performance’ and ‘car’ packages [94,95]. All GLMM models met model distribution assumptions. To test the assumptions of mixed effect Cox models, we quantified the relationship between scaled Schoenfeld residuals and time (‘finalfit’ package) [96]. The proportional hazard assumptions were met for all Cox models. Test statistics were calculated using likelihood ratio tests (‘stats’ package) [89] for GLMM models and Cox models (‘performance’ package) [94]. The residual degree of freedom for GLMM models and hazard ratios for Cox models were derived from model summaries (‘base’ package) [89]. The ‘emmeans’ and ‘multcomp’ packages [97,98] were used to derive means and standard errors of response variables from GLMM models. Figures were made using ‘ggplot2’, ‘ggsignif’, ‘ggsurvfit’ and ‘survival’ packages [92,99–101].

Detailed descriptions of models, error distributions and R syntax can be found in electronic supplementary material, table S1, with brief descriptions provided below. GLMM models were used to test the effect of host plant temperature exposure on number of open flowers (blueberry, phacelia, clover), number of flowers visited (blueberry, phacelia, clover), number of eggs laid, mass of pollen collected per provision, larval development duration, pre-winter cocoon weights, post-winter cocoon weights, pupal weight lost after overwintering, adult emergence timing, adult longevity, adult intertegular distance (ITD) and adult body weight. Cox models were used to test the effect of host plant temperature exposure on the proportion of larval survival, proportion of adult emergence and proportion of adult survival. Random effects were included in all models to account for variation inherent in the experimental design (e.g. field cage identity, block identity, plant identity, mother bee identity, well plate identity) and in observation (e.g. observation date and time) (see electronic supplementary material, table S1 for details). When appropriate, a unique identifier for mother bee identity was created using the ‘dplyr’ package [102]. For analysis of the number of eggs laid, we ran two separate models. The first model excluded all bees who failed to produce eggs, testing whether temperature treatment affected fecundity of individuals successfully initiating egg laying. The second model excluded all bees who lived <6 days, as these bees did not live long enough to begin egg laying, but otherwise included all bees.

## 3. Results

### (a) Bee behaviour

**Figure 1.**
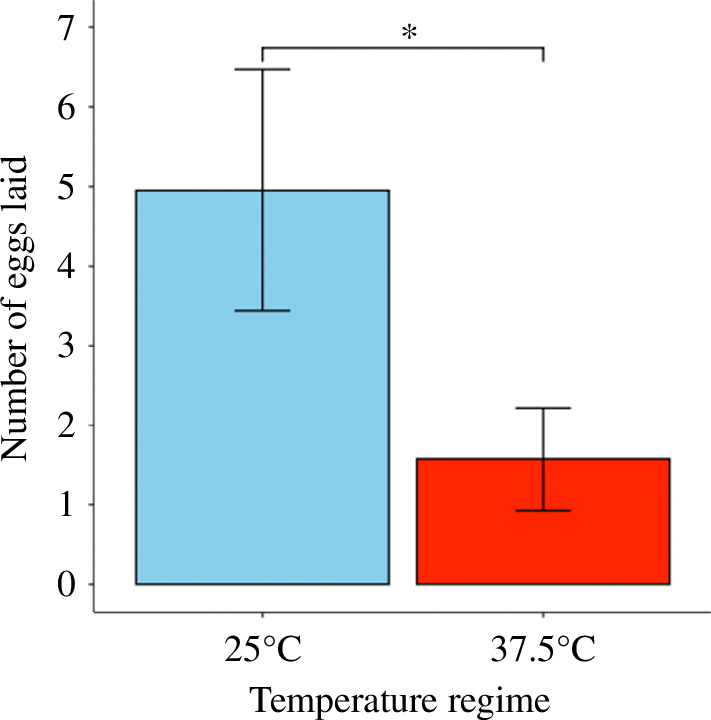
The mean (±s.e.) number of eggs laid by *Osmia lignaria* provided with host plants (blueberry, phacelia and clover) exposed to CT (25°C for 4 h) or HT (37.5°C for 4 h) treatments. The significant difference between means is indicated by the asterisk above error bars (‘*’ = *p ≤* 0.05). *p*-value indicating significance derived from a likelihood ratio test (LRT) of GLMM model.

For each host plant type (blueberry, phacelia, clover), we found no significant relationship between temperature treatment (CT versus HT) and the number of open flowers, or the number of flowers visited by female bees (Electronic supplementary material, table S2). We also found no significant relationship between host plant temperature exposure and the average pollen mass per provision collected by female bees (*χ*² = 0.94, d.f. = 1,35, *p *= 0.33). The mean (±s.e.) pollen provision mass collected by females provided with CT plants was 0.12 ± 0.09 g compared to 0.06 ± 0.02 g by females provided with HT plants. Host plant temperature treatment was significantly associated with female fecundity, both for females that successfully initiated egg laying (figure 1; *χ*² = 4.20, d.f. = 1,7, *p* = 0.04) and for female bees who lived in cages for more than six days (*χ*² = 7.02, d.f. = 1,13, *p *= 0.008). For bees who successfully initiated egg laying, the mean number of eggs laid was significantly lower (68%) in HT cages (1.57 ± 0.65) compared to CT cages (4.95 ± 1.51). For female bees who lived in cages for >6 days, we also found significantly fewer eggs laid (78%) in HT cages (0.68 ± 0.30) compared to CT cages (3.08 ± 1.11). We released 16 bees in HT cages, and 10 bees (62.5%) failed to produce eggs. For CT cages, 12 bees were released and 4 bees (33.3%) failed to produce eggs. In the HT cages, 5 bees who lived *>*6 days failed to lay eggs (31.3%) and 1 bee in CT cages lived *>*6 days and failed to lay eggs (8.3%). In total, females in cages with CT plants laid 33 eggs while those in cages with HT plants laid 10 eggs, a 70% decrease in total egg production.

### (b) Larval development and survival

**Figure 2.**
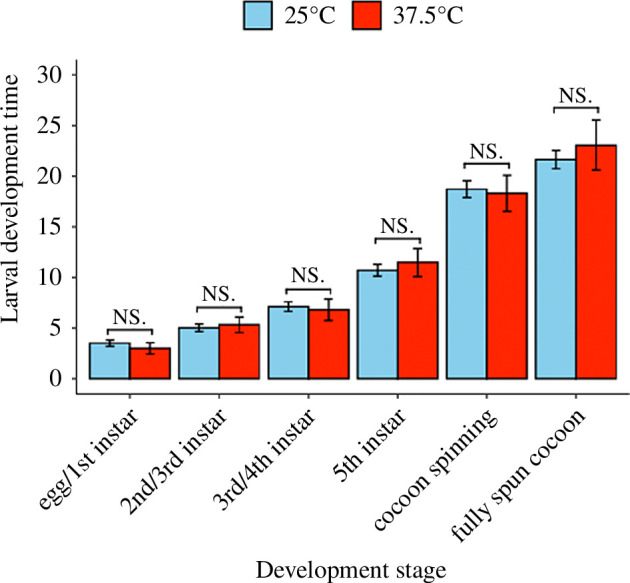
The mean (±s.e.) larval development timing (days) of *Osmia lignaria* larvae fed pollen from host plants exposed to CT (25°C for 4 h) or HT (37.5°C for 4 h) treatments. *p*-values derived from the likelihood ratio test (LRT) of GLMM model showed no significant difference in development timing between temperature treatments, for each development stage, indicated by ‘NS’ above error bars.

**Figure 3.**
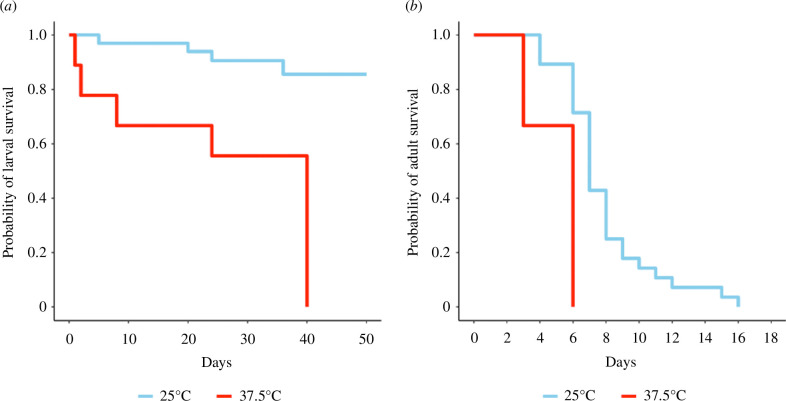
Kaplan–Meier survival probability curve of *Osmia lignaria* fed pollen (as larvae) from host plants exposed to CT (25°C for 4 h) or HT (37.5°C for 4 h) treatments during (*a*) larval development and (*b*) adulthood.

Host plant temperature exposure was not significantly associated with the duration of larval development across all development stages (figure 2: *χ*² = 1.33, d.f. = 2,208, *p* = 0.93). In contrast, larval survival was significantly correlated with host plant temperature exposure (figure 3*a*: *χ*² = 17.83, d.f. = 1, *p* = 0.002, number of events = 42). Larvae consuming pollen from HT plants were 8.26 times more likely to die compared to larvae consuming pollen from CT plants (coef = 2.11, exp(coef) = 8.26, s.e.(coef) = 0.92). In CT cages, 29 of the 33 eggs laid survived to pupation, resulting in 12% larval mortality, whereas in HT cages, 4 of the 10 eggs laid survived to pupation, resulting in 60% larval mortality. Host plant temperature treatment was not significantly associated with pre-winter weight of pupae (*χ*² = 0.06, d.f. = 1,28, *p* = 0.80; CT: 0.053 ± 0.009 g; HT: 0.055 ± 0.012 g), post-winter weight of pupae (*χ*² = 0.31, d.f. = 1,27, *p* = 0.58; CT: 0.045 ± 0.005 g; HT: 0.048 ± 0.010 g) or weight lost during overwintering (*χ*² = 0.28, d.f. = 1,27, *p* = 0.60; CT: 0.016 ± 0.003 g; HT: 0.014 ± 0.003 g).

### (c) Adult emergence, body size and survival

Adult bee emergence was significantly correlated with host plant temperature exposure (*χ*² = 10.59, d.f. = 1, *p* = 0.035, number of events = 33). Progeny fed with HT pollen as larvae were 50% less likely to emerge as adults compared to progeny fed with CT pollen (coef = −0.69, exp(coef) = 0.50, s.e.(coef) = 0.78). The timing of adult emergence was not significantly associated with host plant temperature exposure (*χ*² = 1.02, d.f. = 1,25, *p* = 0.31). The mean (±s.e.) days until emergence was 10.64 ± 2.36 for bees consuming HT pollen and 8.45 ± 0.65 for bees consuming CT pollen. Adult body weight (*χ*² = 0.09, d.f. = 1,25, *p* = 0.77) and ITD (*χ*² = 0.25, d.f. = 1,25, *p* = 0.61) were not significantly associated with host plant temperature exposure. The mean (±s.e.) adult body weight and ITD (respectively) for progeny fed with HT pollen as larvae was 0.037 ± 0.009 g and 2.71 ± 0.22 mm, compared with 0.035 ± 0.005 g and 2.58 ± 0.16 mm for those who consumed CT pollen as larvae. Adult bee survival had a significant relationship with host plant treatment (figure 3*b*: *χ*² = 21.39, d.f. = 1, *p* = 0.001, number of events = 31). Progeny provided with HT pollen provisions as larvae were 3.54 times more likely to die as adults compared to progeny provided with CT pollen (coef = 1.26, exp(coef) = 3.54, s.e.(coef) = 0.77). The longevity of adult survival was not significantly associated with host plant temperature (*χ*² = 1.37, d.f. = 1,26, *p* = 0.24). Bees fed HT pollen as larvae lived 5.39 ± 1.56 days as adults and bees fed CT pollen lived 7.66 ± 0.74 days as adults. Far fewer progeny survived into adulthood when provided with pollen from HT plants. Of the 10 eggs laid by females provided with HT plants, only 3 emerged as adults (70% mortality from egg to adulthood) compared to the bees provided with CT plants where 28 of the 33 eggs laid emerged as adults (15% mortality).

## 4. Discussion

Anthropogenic climate change has caused substantial damage to ecosystems across the globe, and extreme heat events are a main driver for these changes [5]. Despite the importance of insect pollinators for ecosystem health and global agriculture, we know relatively little about the current and long-term effects of extreme heat on pollinator health, productivity, functioning and interactions with other organisms. Increasingly, researchers are considering the indirect impacts of climate warming on bees by evaluating changes in floral rewards and bee behaviour [28,29,41,103], but no studies to our knowledge have evaluated how field-realistic acute extreme heat exposure to host plants affects mother bees and their offspring. Our investigation into the indirect effects of extreme heat provides the first evidence of adverse consequences for solitary bees and their brood, mediated through their host plants. Female *O. lignaria* foraging on plants exposed to only 4 h of extreme heat during bloom laid significantly fewer eggs, and the majority of larvae consuming heat-stressed pollen died before pupation. The offspring who consumed heat-stressed pollen as larvae and pupated had lower emergence and greater risk of mortality as adults. We also observed trends for delayed adult emergence and shorter adult lifespan compared to those provided with pollen from non-stressed plants. These results highlight that even brief periods of extreme heat on host plants can have detrimental repercussions for foraging bees and their offspring, expanding our understanding of the implications of climate change for plant–pollinator interactions beyond the direct negative effects [7,104].

The number of open flowers and flower visitation rates were similar between the two temperature groups in this study, indicating consistency of resource availability and resource use across the treatments. Despite this, we observed considerably lower egg laying by bees foraging on heat-stressed plants. This suggests that floral rewards from heat-stressed plants were of lower quantity and/or quality compared to non-stressed plants. In a related study, exposing host plants to heat wave conditions (35/22°C) for three days resulted in 70% lower nectar production compared to plants developing at 25°C, yet the number of flowers visited by bumble bees was the same between treatments [28]. Other studies have found lower pollen production and release following heat exposure [24,29,43,105]. Our finding of similar-sized pollen provisions between treatments, yet fewer eggs laid in the heat-stressed treatments, may reflect reduced availability of floral resources from individual flowers. Thus, bees provided with heat-stressed plants may have needed greater foraging efforts to collect enough pollen for greater brood production. However, other studies have reported similar amounts of pollen produced from stressed- and non-stressed plants, yet the viability of pollen is lower, which may affect the quality of the diet provided to bees [10,103]. Additional studies are needed to measure changes in pollen production and composition following heat stress, and subsequent effects on pollinator visitation and plant reproduction.

Reduced egg laying by bees provided with heat-stressed plants may also be attributed to an insufficient diet adversely affecting their reproduction. Egg production and oviposition are energetically costly for all bees, but particularly for solitary bees that lay large eggs relative to their body size and require pollen for oocyte maturation [106]. Other *Osmia* studies have shown that females foraging in low-resource conditions produced fewer brood cells per day [51], and when denied access to pollen, females failed to mature oocytes or lay eggs [106]. *Osmia cornuta* females can produce 40–50 oocytes but rarely lay more than 10–20 eggs, suggesting limitations on fecundity may be attributed to constraints on brood cell provisioning (like reduced resource availability) rather than egg production potential [107]. When brood provisioning is impeded, adjustments in parental investment allocation can occur, where females may limit the number of brood produced but maintain the provision size provided to offspring [107]. This offers additional insight into why females provided with heat-stressed plants produced less brood but retained similar amounts of provisions for those offspring. Under extreme heat conditions, pollen may not only be in short supply but the nutrients present in pollen may also be altered if heat disrupts the sequestration and metabolic transport of nutrients into developing pollen grains [29,38,41,42,108]. Alterations or deficiencies in pollen nutrition can have devastating consequences for bee reproduction. In honey bees, low-protein diets can inhibit ovarian and egg development [109,110] and diets lacking essential amino acids can prevent brood production and development [111–113]. *Bombus terrestris* provided diets deficient in essential amino acids delayed nest initiation and inhibited brood production [114]. Future studies should compare oocyte and brood production in female bees provided optimal, low-quantity, low-quality or heat-stressed pollen diets.

The consequences of heat-altered nutrient availability on pollen performance and fertilization are well studied [14,24,37,38,108], but there is far less research on the subsequent consequences for bee nutrition (but see [14,27]). In the present study, bees consuming pollen from heat-exposed plants had much higher mortality during larval development, despite similar provision sizes compared to those in control cages. While enough pollen may have been provided for larval development, it is possible that heat-stressed provisions lacked certain essential nutrients, inhibiting their survival. Previous research on inadequate pollen nutrition emphasizes the consequences of a poor diet on bee development and survival. For *O. lignaria* larvae fed honey bee-collected pollen, only the highest protein diets supported development to adulthood [111,115]. Other studies have found that carbohydrates, not protein, mediate *Osmia* larval growth and survival to pupation [116]. *Osmia bicornis* and *O. cornuta* larvae failed to develop on *Tanacetum* pollen, which authors suggest is due to insufficient quantity or quality of nutrients [117]. *Osmia cornifrons* larvae failed to develop when fed multifloral and single-source pollen diets, even when these diets had similar protein:lipid ratios as surveyed provisions, suggesting certain micronutrients were lacking and must be present for proper development [118]. Abnormal development was also observed in our study for larvae that consumed heat-stressed pollen, where a third of these larvae spun unusually light-coloured silk and failed to enclose themselves for pupation. *Osmia* cocoons are an important sink for nutrients assimilated and used during larval development, and underdeveloped cocoons may indicate the scarcity of specific elements present in pollen [119]. *Osmia bicornis* larvae fed a single-source pollen diet failed to enclose their cocoon and had high larval mortality, suggesting chronic nutrient deficiency [49]. When fed high quantities of rapeseed pollen, the same species exhibited hindered cocoon development and high male mortality, but when supplemented with additional nutrients, these negative effects were absent [120]. In the context of these other studies, our results suggest a nutritional mechanism for the adverse effects of indirect heat stress on larval development and survival. However, additional research is required to quantify the nutrient composition of heat-stressed pollen to better understand how it affects nutrition and silk production in *Osmia* bees.

We found variable effects of heat-stressed diets on bee development, with higher larval mortality and abnormal cocoon spinning, but no effect on larval development duration. This finding is surprising, but understanding the physiological processes of *Osmia* development may provide insight. Solitary bee larvae provided with pesticide-contaminated diets experience slower development compared to untreated diets, and authors attribute this to detoxification processes that divert time, energy and nutrients away from development [87,121]. In the present study, no detoxification was required as brood were not exposed to pesticides, and the pollen provided came from known host plants, protected from pesticide exposure. Other studies have shown extrinsic cues for *Osmia* development timing and pupation, regulated via starvation and hormone signalling rather than meeting a critical mass as previously assumed [122], possibly providing insight into the similar development timing observed between treatments in our study. There are strong positive correlations between pollen provision mass and weight of *Osmia* cocoons and adults [87,123], and we found no differences in the mass of pollen provisions, cocoons, or adults between treatments. The results indicate similar fat body depletion and respiration rates among surviving brood [85,124–126] and suggest that the indirect effects of extreme heat are primarily mediated through pollen quality affecting progeny survival as larvae and adults.

In spring-emerging bees, like *Osmia*, emergence timing is critical for maximizing resource allocation and fecundity [31,124,127]. When emergence phenologies are altered, individuals may experience lower mating opportunities, foraging potential and fecundity [31,128–130]. In the present study, 50% of bee pupae fed heat-stressed pollen as larvae failed to emerge as adults and took three additional days to emerge compared to the control diet group. Ovary maturation in *O. lignaria* takes 2–3 days, after which females initiate nesting [48]. In another study, *O. lignaria* females who successfully established nests took ~3 days to emerge, while females who failed to establish nests took ~6 days [126]. So, a three-day delay in emergence could have repercussions for *Osmia* egg-laying potential. For males, delayed emergence could impede mating opportunities due to shorter copulation events and heightened reproduction failure, as observed in *O. cornuta* [131]. Delayed emergence is also relevant for crop pollination, where mismatches in bee emergence and bloom could inhibit pollination services [54,55,132,133].

All emerged progeny in this study were males. This could be because the nutrients present in pollen provisions were adequate for male development and survival, but not for females [119]. Higher concentrations of certain nutrients are found in female *Osmia* cocoons, potentially reflecting higher production costs and nutrient demands for female bees than male bees [119]. Alternatively, female bees may preferentially produce progeny of the smaller sex (males) when floral resources are limited [107]. Given our limited sample size in this study, future research should consider scaling up this design in the field or exploring these mechanisms in laboratory settings. Nonetheless, our study is among the first to provide strong evidence that brief periods of extreme heat can indirectly reduce female fecundity and subsequent offspring survival.

The longevity of *Osmia lignaria* adults in the absence of food reflects the quality of diet received as larvae and survival under natural conditions when food sources are limited [59,133]. Bees fed heat-stressed pollen as larvae had a greater risk of mortality and shorter lifespans (~3 days) as adults compared to those fed non-stressed pollen. Considering emergence delays and reduced lifespans, bees fed heat-stressed pollen would be active for ~6 fewer days compared to offspring fed non-stressed pollen. *Osmia lignaria* are typically active for 20–30 days during the spring [48], so this could result in a 22–35% reduction in adult lifespan. Female *O. lignaria* can complete 1–2 provisioned brood cells per day [134] or 20–60 brood cells throughout their lifetime, so six fewer days of activity could limit brood production, considering the strong relationship between fecundity and longevity in *Osmia* [127]. These bees are already in decline in certain regions of the US [56], so increasing constraints on their fecundity and longevity may further perpetuate these patterns. It is also important to highlight that the bees in this study were reared and maintained under optimal conditions, so negative consequences are likely to be amplified if adult bees and their offspring are directly exposed to heat stress or other stressors including resource limitation, pesticide exposure and pathogens.

## 5. Conclusions

Our study highlights that the indirect effects of climate change and extreme heat can have cumulative impacts on bees, their offspring and their host plants. Furthermore, our results suggest that studies evaluating direct heat stress on bees may underestimate the consequences of climate change on bee–plant interactions. There is an urgent need for mitigation strategies to help protect host plants, and in return, help protect bees such as overhead irrigation to cool fields during bouts of extreme heat [135,136] which may protect pollen and its nutritional quality. Combating the effects of climate change-induced heat extremes will require creative solutions on both short- and long-term scales. To effectively address the implications of heat stress on bees and their interactions, we must broaden our understanding from direct, isolated stressors to indirect and interactive stressors. Our results demonstrate that the indirect effects of extreme heat on bee physiology and development should be further explored to better understand the implications for bee populations, wild plants and agricultural production.

## Data Availability

All data and R code used in this study can be accessed at Figshare [137]. Supplementary material is available online [138].
